# Assessment of the MANTA closure device in transfemoral transcatheter aortic valve replacement: a single-centre observational study

**DOI:** 10.1007/s12471-020-01465-3

**Published:** 2020-07-27

**Authors:** J. Halim, L. Missault, M. Lycke, J. Van der Heyden

**Affiliations:** Department of Cardiology, Sint-Jan Hospital, Bruges, Belgium

**Keywords:** Vascular access, Complication, Transcatheter aortic valve replacement, Vascular closure device

## Abstract

**Objectives:**

The present study aims to evaluate the efficacy and safety of the MANTA vascular closure device (VCD) (Teleflex, Morrisville, NC, USA) in transfemoral transcatheter aortic valve replacement (TF-TAVR).

**Background:**

To close the femoral artery in TF-TAVR a VCD is the treatment of choice. Data involving suture-based VCDs have been extensive. Although scarce, results on the MANTA device are promising. There is no consensus yet as to whether the MANTA device is associated with fewer access-site-related vascular/bleeding complications when compared to suture-based VCDs.

**Methods:**

In this prospective single-arm study, performed at a single centre, a total of 73 patients eligible for TF-TAVR were included and consecutively treated with the MANTA device.

**Results:**

Access-site-related vascular complications were seen in 13.7% of patients treated with the MANTA device. In this group of patients only minor vascular complications were observed. Access-site-related bleeding complications were rare (6.8%), and device failure was seen in 13.7% of the patients.

**Conclusions:**

This single-centre study confirms that use of the MANTA device in TF-TAVR is feasible with an acceptable rate of access-site-related complications and no major vascular complications.

## What’s new

- Access-site-related vascular and bleeding complications in transfemoral transcatheter aortic valve replacement (TF-TAVR) remain a problem with a significant impact on clinical outcomes.
- The MANTA vascular closure device (VCD) is efficacious and safe for use in TF-TAVR. Importantly, major vascular complications did not occur and major bleeding complications were extremely rare when using this device.
- A randomised controlled trial comparing the MANTA device with suture-based VCDs is needed to determine which device is associated with the lowest risk of access-site-related complications.

## Introduction

Transcatheter aortic valve replacement (TAVR) has emerged as the gold standard treatment for symptomatic patients at high surgical risk. While the complication rate has decreased as a result of the operators’ growing expertise and knowledge, as well as new valve technology, TAVR is still associated with a significant complication rate [1].

More specifically, vascular complications at the access site for transfemoral (TF)-TAVR still remain a problem. These complications are observed in 5–20% of patients and have a significant impact on clinical outcomes [2]. During the early years of TF-TAVR, the femoral artery was closed surgically. More recently, this method has been replaced by use of a vascular closure device (VCD).

The most commonly used VCDs for TF-TAVR are suture-based, e.g. the ProGlide and the Prostar XL (Abbott Vascular Inc., Santa Clara, CA, USA). The efficacy of these VCDs has been extensively reported in previous studies [3]. The MANTA VCD (Teleflex, Morrisville, NC, USA) is a collagen-based device that has shown promising results in TF-TAVR [4–9]. However, data regarding the vascular complication rate in comparison to the well-known suture-based VCDs are scarce.

The primary aim of this study is to assess access-site-related complications in TF-TAVR patients treated by the MANTA device. Our hypothesis is that the MANTA device is associated with a low risk of major vascular and major bleeding complications according to the Valve Academic Research Consortium (VARC)-2 definitions [10].

## Methods

### Study design

A total of 73 patients with severe aortic stenosis scheduled for TF-TAVR were consecutively included between July 2018 and August 2019 at the Sint-Jan Hospital in Bruges, Belgium. Informed consent was obtained in accordance with the Declaration of Helsinki. Multi-detector computed tomography was performed according to the TAVR protocol, using dedicated software (3mensio, Pie Medical Imaging, Maastricht, The Netherlands). Initially, suture-based VCDs were used at our centre. However, since July 2018 the MANTA device has been utilised. Starting then and for the purposes of this trial, data from patients treated with the MANTA device were collected prospectively. Two TAVR operators with prior experience in using the MANTA device performed the vascular closure.

### Procedure details

In patients not taking antiplatelet medication, acetylsalicylic acid was started prior to the procedure. Additional clopidogrel was started on the morning of the procedure. Novel oral anticoagulant therapy was discontinued 24 h before the procedure. Oral anticoagulant therapy was continued, aiming for an International Normalised Ratio level of 2–2.5.

All procedures were performed with the patient under conscious sedation. Puncture of the femoral artery was performed with either fluoroscopic or ultrasound guidance. During the procedure, heparin was given and an activated clotting time between 250 and 300 s was targeted. The implanted bioprosthetic valves were either the CoreValve Evolut R/PRO (Medtronic, Minneapolis, MN, USA), the ACURATE neo (Boston Scientific, Natick, MA, USA) or the LOTUS Edge (Boston Scientific). In patients treated with a CoreValve a 16-French (Fr) InLine sheath or a 20-Fr Sentrant introducer sheath was used. In patients treated with an ACURATE neo a 14-Fr iSLEEVE or a small LOTUS introducer sheath was used. A large LOTUS introducer sheath was used for implantation of the LOTUS Edge. After valve implantation, in cases where the activated clotting time was above 200 s, protamine was given before vascular closure.

### MANTA device

The MANTA closure device is a collagen-based technology available in two sizes: 14 Fr and 18 Fr. It can be used for sheath sizes ranging from 10 to 14 Fr and from 15 to 22 Fr respectively. A detailed description of the MANTA device has been published previously [11]. In summary, it consists of multiple components: an 8‑Fr puncture site dilator, a dedicated sheath, a closure unit and a delivery system. The site dilator and device sheath have centimetre markers. After successfully puncturing the femoral artery, a standard 6‑Fr sheath is removed and the site dilator is pushed over the guidewire to measure the distance from skin level to the endoluminal space of the vessel. When vascular closure is initiated, the access sheath is removed and replaced by the MANTA sheath. The closure unit is connected with the MANTA sheath and both are withdrawn up to the predetermined deployment level. The toggle is then released, and the device is withdrawn with the colour code of the tension gauge serving as an indicator of whether sufficient pulling force is being applied. A blue tamper tube is then advanced along the suture line to position the radiopaque lock over the suture knot to secure the collagen-anchor sandwich. When haemostasis has been achieved the guidewire is removed and the suture is cut at skin level. Importantly, unlike the suture-based VCDs a pre-closure technique is not necessary [12]. Further, it may take 6 months until the components of the MANTA device are resorbed.

### Femoral artery evaluation: accessibility and closure device success

Multi-detector computed tomography was performed to assess the iliofemoral arteries for vascular access. The vessel size, tortuosity, the amount of calcification and the minimum lumen diameter were analysed. Notably, a sheath to femoral artery ratio of ≥1.05 is associated with a higher risk of vascular complications and 30-day mortality [13, 14].

Haemostasis success after use of the MANTA device was evaluated by use of rotational angiography after vascular closure. This meant a dynamic evaluation of the closure site from the 30-degree right anterior oblique view to the 30-degree left anterior oblique view with administration of 20 cc contrast medium. If leakage was present it could be classified as mild (minor extravasation of contrast without external bleeding), moderate (continuous extravasation with moderate external bleeding) or severe (continuous extravasation with significant external bleeding). If leakage occurred balloon dilatation was performed. Hereafter, rotational angiography was repeated and if leakage persisted a covered Gore Viabahn stent (Gore Medical, Flagstaff, AZ, USA) was placed.

### Study endpoints

The primary endpoint of this study was the evaluation of device failure and vascular/bleeding complications at the access site according to the VARC‑2 definitions. Secondary endpoints included mortality and vascular/bleeding complications at the access site at 30-day follow-up.

### Statistical analysis

Statistical analysis included descriptive statistics. Categorical variables were presented as frequencies and percentages and continuous variables as mean ± standard deviation (SD). All analyses were conducted with SPSS v.26 (IBM, Chicago, IL, USA).

## Results

### Baseline characteristics

A total of 73 patients were included in our study and treated with the MANTA device. Baseline characteristics are shown in Tab. 1. The mean age of this group of patients was 84 ± 5.48 years and the majority of these patients were men (54.8%). Antiplatelet therapy was used in more than half of the patients. Evolut R/PRO was implanted in more than half of the cases (64.4%).

**Table 1:** Baseline characteristics

	**(*n*** **=** **73)**
*Age (years)*	84.0 ± 5.48
*Men*	40 (54.8)
*BMI*	26.4 ± 4.25
*NYHA 3 or 4*	24 (32.9)
*Euroscore II*	4.6 ± 3.79
*LVEF <40%*	5 (6.8)
*Aortic valve MG*	43.1 ± 15.76
*AVA*	0.73 ± 0.20
*Antiplatelet therapy*	42 (57.5)
*Anticoagulant therapy*	23 (31.5)
**Femoral artery characteristics**	
*Minimum arterial diameter (MANTA side) (mm)*	7.2 ± 1.49
*Calcification (MANTA side):*	
*–* None	26 (35.6)
*–* Mild	32 (43.9)
*–* Moderate	9 (12.3)
*–* Severe	6 (8.2)
**Procedural characteristics**	
*Device system:*	
*– Evolut PRO*	
*–* 23 mm	1 (1.4)
*–* 26 mm	7 (9.6)
*–* 29 mm	16 (21.9)
*– Evolut R*	
*–* 34 mm	23 (31.5)
*– ACURATE neo*	
*–* small	2 (2.7)
*–* medium	6 (8.2)
*–* large	17 (23.3)
*– Lotus Edge*	
*–* 27 mm	1 (1.4)
*MANTA size:*	
*–* 14 Fr	0 (0)
*–* 18 Fr	73 (100)
*Femoral sheaths:*	
*–* 14 Fr, iSLEEVE	13 (17.8)
*–* 16 Fr, InLine sheath	45 (61.6)
*–* 20 Fr, Sentrant sheath	3 (4.1)
*–* Small LOTUS	11 (15.1)
*–* Large LOTUS	1 (1.4)
*Sheath to femoral artery ratio*	1.03 ± 0.27

Data presented as number (%) or mean ± SD

*SD* standard deviation, *BMI* body mass index, *NYHA* New York Heart Association, *LVEF* left ventricular ejection fraction, *MG* mean gradient, *AVA* aortic valve area, *Fr* French

Mean minimal femoral arterial diameter at the access site was 7.2 ± 1.49 mm and mean sheath to femoral artery ratio was 1.03 ± 0.27. In all patients the MANTA 18-Fr device was used for femoral artery closure.

### Outcomes

#### Primary endpoint

Outcomes of device failure and vascular/bleeding complications at the access site are shown in Tab. 2. Ten patients (13.7%) had device failure. In these 10 patients use of a covered Gore Viabahn stent was necessary. Details of the femoral artery and introducer sheath used in this group of patients are summarised in Tab. 3. No major differences could be seen when compared to the femoral artery and introducer sheath characteristics of the entire study population.

**Table 2:** Primary and secondary endpoints

	(*n* = 73)
Device failure	10 (13.7)
All vascular complications (major + minor)	10 (13.7)
Major vascular complications	0 (0)
Minor vascular complications	10 (13.7)
Surgical closure	0 (0)
All bleeding complications (major + minor)	5 (6.8)
Major bleeding complications	1 (1.4)
Minor bleeding complications	4 (5.5)
30-day mortality	4 (5.5)
30-day vascular/bleeding complications	1 (1.4)

Data presented as number (%)

**Table 3:** Vessel and sheath characteristics of patients with device failure

	(*n* = 10)
**Femoral artery characteristics**	
*Minimum arterial diameter (MANTA side) (mm)*	7.04 ± 0.71
*Calcification (MANTA side)*	
– None	5 (50)
– Mild	4 (40)
– Moderate	1 (10)
**Procedural characteristics**	
*Femoral sheaths:*	
– 14 Fr, iSLEEVE	5 (50)
– 16 Fr, InLine sheath	3 (30)
– Small Lotus	2 (20)
*Sheath to femoral artery ratio*	1.08 ± 0.18

Data presented as number (%) or mean ± SD

*Fr* French

Vascular complications were present in 10 patients (13.7%). However, major vascular complications did not occur. Minor vascular complications were present in 10 patients (13.7%), in whom balloon dilatation did not result in haemostasis; subsequently, covered stent implantation was performed. Additional surgery was not needed.

Bleeding complications occurred in five patients (6.8%), but none were life-threatening or disabling. One patient experienced a major bleeding complication at the groin, resulting in a drop in haemoglobin of greater than 3.0 g/dl and the need for a blood cell transfusion. A minor bleeding complication was present in four patients, in whom prolonged manual compression led to an access site haematoma and a slight fall in haemoglobin level.

#### Secondary endpoint

Late major bleeding complications were seen in one patient at 30-day follow-up. In this patient a haematoma at the femoral access site as well as haematuria occurred. Three units of packed red blood cells were transfused. Late vascular complications did not occur.

Thirty-day mortality was 5.5% (*n* = 4). One patient developed cardiogenic shock during hospitalisation, which led to cardiac arrest. Echocardiography in the acute setting did not reveal structural abnormalities. The second patient underwent a valve-in-valve intervention (previous Solo stentless bioprosthetic aortic valve implantation; LivaNova, London, UK), whereby implantation of the new bioprosthetic valve led to occlusion of the left main coronary artery, resulting in cardiac arrest and death. The third patient experienced a periprocedural myocardial infarction. Shortly hereafter, cardiogenic shock resulted in death. Lastly, one patient died following complications resulting from a periprocedural cerebrovascular accident.

## Discussion

In this prospective single-centre study evaluating the MANTA device in TF-TAVR, no major vascular complications were encountered. A major bleeding complication occurred in one patient (1.4%). Minor vascular and minor bleeding complications were seen in 13.7% and 5.5% of the patients respectively. Device failure occurred in 13.7% of the patients. At 30-day follow-up, mortality and the rate of vascular/bleeding complications were low.

Van Mieghem et al. [4] performed a single-arm prospective study including 50 patients, in whom the use of the MANTA device in TF-TAVR and high-risk percutaneous coronary intervention was associated with rapid haemostasis success and a low vascular complication rate. Major vascular and major bleeding complications were each seen in 2% of the patients. Minor vascular complications were not observed. De Palma et al. [5] evaluated the MANTA device in TF-TAVR in a prospective observational study and revealed similarly promising results with major vascular and major bleeding complications each being present in only 1.1% of the patients. Minor vascular and minor bleeding complications occurred in 4.5% and 11.2% of the patients respectively. Wood et al. [6] assessed the MANTA device prospectively in 341 patients undergoing TAVR or endovascular aneurysm repair and noted rapid haemostasis success and a low rate of major vascular and major bleeding complications (2.3% and 1.1% respectively). VARC-2-defined minor vascular and minor bleeding complications were not reported. In the study of Biancari et al. [7], higher rates of major vascular and major bleeding complications (9.3% and 15.9%) were observed. Minor vascular complications were present in 3.7% of their patients. Minor bleeding complications were not noted. Moriyama et al. [8] reported vascular complications in 8% of their patients. Major and minor bleeding complications were seen in 10% and 4% of the patients. Moccetti et al. [9] also reported acceptable rates of major vascular and major bleeding complications (9% and 3% respectively) when using the MANTA device: 5% of the patients had a minor vascular complication and 7% of the patients a minor bleeding complication. Importantly, MANTA-associated vascular complications were seen more often in patients with significant peripheral disease and narrower (<6 mm) femoral arteries.

To date, there is no consensus as to whether the MANTA device is associated with fewer VARC‑2 access-site-related major vascular/bleeding complications when compared to the suture-based VCDs. Biancari et al. [7] retrospectively compared the MANTA device with the ProGlide device in 222 patients. There were no significant differences in VARC‑2 vascular and bleeding complications between these two devices. However, in the study by De Palma et al., fewer major bleeding complications were seen with the MANTA device than with the Prostar XL device [5]. In the above-mentioned trial by Moriyama et al., including 325 patients, these two VCDs were compared retrospectively by use of a propensity-matched analysis. In the MANTA group, there were significantly fewer bleeding complications—importantly, mainly as a result of the lower number of major bleeding complications. Access-site-related and access-related vascular injuries were also observed less frequently in the MANTA group. The incidence of major vascular complications was, however, comparable between these two groups [8].

The present study revealed a similar trend towards a low rate of access-site-related major vascular and major bleeding complications when using the MANTA device. No major vascular complications were observed.

The presence of minor vascular complications was slightly higher than has been reported previously. In the study of Van Mieghem et al. [4], for instance, where iliofemoral angiography was performed after vascular closure, minor vascular complications were not encountered and only three patients had signs of contrast extravasation. However, it should be noted that in the studies of De Palma et al. and Biancari et al. iliofemoral angiography was not performed systematically after vascular closure, and this was not mentioned in the study of Moriyama et al. This could be a possible explanation for the differences in minor vascular complications reported in these studies.

In addition, in the present study haemostasis success was consistently determined by rotational angiography in contrast with other studies where this was done by external inspection. A covered stent was placed due to persistent leakage without the presence of a major bleeding or signs of visceral ischaemia. Hence, a lower threshold for the use of balloon dilatation and covered stent placement could have been set compared to that in other studies.

The prevalence of vascular and bleeding complications in this study was largely comparable with that reported in previous studies and confirms that the MANTA device is a feasible option in TF-TAVR patients [2, 15]. Notably, a low rate of major access-site-related complications was observed. The MANTA device shows promising results and as it demands a shorter learning curve in comparison with the suture-based VCDs there is a possibility that the MANTA device will become the preferred option for vascular closure in TF-TAVR. However, future randomised controlled studies comparing the MANTA device with the suture-based VCDs represent a necessary first step to elucidate which type of VCD is associated with the lowest risk of access-site-related complications in TF-TAVR patients.

### Limitations

This study has several limitations. First, the sample size is relatively small and the study has a non-randomised design. Second, the performance of the 14-Fr MANTA device cannot be assessed because the 18-Fr device was used in all patients undergoing TF-TAVR. However, there was no selection bias, with the enrolment of consecutive patients treated with the MANTA device.

## Conclusion

The use of the MANTA device, which is a collagen-based VCD, is feasible in TF-TAVR patients and is associated with acceptable rates of vascular and bleeding complications at the access site. One major bleeding complication and no major vascular complications were seen after use of the MANTA device.
